# Assessing the contribution of immigrants to Canada’s nursing and health care support occupations: a multi-scalar analysis

**DOI:** 10.1186/s12960-022-00748-7

**Published:** 2022-06-13

**Authors:** Rafael Harun, Margaret Walton-Roberts

**Affiliations:** 1 Department of Social Work and Urban Studies, Tennessee State University, Nashville, TN USA; 2 Balsillie School of International Affairs, Wilfrid Laurier University, Waterloo, ON Canada

**Keywords:** Internationally educated nurses, Canada, Workforce planning, Immigration

## Abstract

**Background:**

The World Health Organization adopted the Global Strategy on Human Resources for Health Workforce 2030 in May 2016. It sets specific milestones for improving health workforce planning in member countries, such as developing a health workforce registry by 2020 and ensuring workforce self-sufficiency by halving dependency on foreign-trained health professionals. Canada falls short in achieving these milestones due to the absence of such a registry and a poor understanding of immigrants in the health workforce, particularly nursing and healthcare support occupations. This paper provides a multiscale (Canada, Ontario, and Ontario’s Local Health Integration Networks) overview of immigrant participation in nursing and health care support occupations, discusses associated enumeration challenges, and the implications for health workforce planning focusing on immigrants.

**Methods:**

Descriptive data analysis was performed on Canadian Institute for Health Information dataset for 2010 to 2020, and 2016 Canadian Census and other relevant data sources.

**Results:**

The distribution of nurses in Canada, Ontario, and Ontario’s Local Health Integration Networks reveal a growth in Nurse Practitioners and Registered/Licensed Practical Nurses, and contraction in the share of Registered Nurses. Immigrant entry into the profession was primarily through the practical nurse cadre. Mid-sized communities registered the highest growth in the share of internationally educated nurses. Data also pointed towards the underutilization of immigrants in regulated nursing and health occupations.

**Conclusion:**

Immigrants comprise an important share of Canada’s nursing and health care support workforce. Immigrant pathways for entering nursing occupations are complex and difficult to accurately enumerate. This paper recommends the creation of an integrated health workforce dataset, including information about immigrant health workers, for both effective national workforce planning and for assessing Canada’s role in global health workforce distribution and utilization.

## Background

In response to United Nation’s third Sustainable Development Goal (SDG 3) of ensuring healthy lives and to promote well-being for all at all ages, the WHO adopted the Global Strategy on Human Resources for Health: Workforce 2030 in May 2016 [26, 35]. The strategy set some global milestones. For example, by 2020, it is expected that member countries would make progress on developing a health workforce registry to track stocks, distribution, demand, and supply. By 2030, member countries are also expected to make progress towards self-sufficiency by halving their dependency on foreign-trained health professionals. As outlined in the WHO Global Code of Practice on the International Recruitment of Health Personnel [34], this will also contribute to overall efforts to protect health systems in lower income nations where immigrant health workers tend to originate.

At present, Canada and its provinces are yet to achieve these milestones. There is no universal registry of health workers in Canada recording stock, demand, and supply [7]. Canadian Institute for Health Information (CIHI) does provide information on health human resources (HRH) in six regulated professions (nurses, occupational therapists, pharmacists, physicians, physiotherapists), but data functionality and timeliness need to be improved, there is no data on forecasted supply, unregulated providers are not included, pan-Canadian comparisons across occupational cadres are difficult to assess, and the ability to review data at different scales (regional, local health units, etc.) is constrained [8]. Socio-demographic data on HRH are also limited, particularly in terms of ethnicity and citizenship, which makes measuring the contribution of immigrants to the Canadian health workforce difficult. This is a significant problem, since assessing the contribution of internationally educated health care workers to Canada’s health workforce is necessary to address the goals of the WHO’s Global Strategy self-sufficiency milestone, as well as understand how well Canada is abiding by the spirit of the WHO Code.

Health workforce planning in Canada warrants a comprehensive understanding of immigrant participation in health and care related services because immigration is a key Canadian social policy and plays a central role in labour force growth [14]. Landed immigrants aged between 25 and 54 years accounted for nearly 60 percent of the country’s employment gains in 2017 [37]. This is particularly true for nursing and care-related services. Nearly 21 percent of the total employed workers in nursing and health care support occupations in Canada are immigrants, and the proportion is growing [12, 37]. The number of Internationally Educated Nurses (IENs) in Canada’s nursing workforce has increased from 6.9 percent (23,764) in 2007 to 9 percent (37,370) in 2019 [8, 9]. Also, between 1996 and 2016, the proportion of immigrants employed as nursing aides has grown by 14 percent, whereas the corresponding proportion in all other occupations has increased by only 5 percent [31].

Despite these figures, there is a paucity of data regarding the potential number of immigrants who could work in the health sector [4–6]. During the COVID-19 global pandemic employers recognized the need to improve their understanding of current and potential supply of immigrants for the nursing and health care support occupations, as well as the need for better planning to optimize immigrant integration into the Canadian health workforce [36].

The objective of this article is to provide a multi-scalar review of immigrants’ employment in nursing and health care support occupations in Canada and Ontario to identify lessons learned in the development and implementation of HRH policies and programmes. The paper is situated at the intersection of the World Health Organization’s (WHO) call for a health workforce registry for systematic health work force planning, and the recurring shortage of nursing and related care providers in the Canadian case, which is partly addressed through the incorporation of immigrants with various levels of prior health training. We also recognize that we need to better understand Canada’s immigration process with regard to health workers and how it might affect adherence to the spirit of the WHO Code.

The article is structured as follows. We provide background on the pathways that immigrants follow to enter the nursing and care support services in Canada, and highlight some of the associated data challenges to assessing this phenomenon. We then discuss our methodology and using available datasets we explore the present level of immigrant participation in nursing and care occupations (both regulated and unregulated) in Canada, Ontario, and specific Local Health Integration Networks (LHINs).^1^ We also consider the contribution immigrants make in unregulated care aide positions. Finally, reflecting the WHO Global Strategy, we recommend future steps for improved health workforce planning that account for the scale and contribution of immigrant health care workers.

## Immigrant pathways into nursing and health care support occupations and related data challenges

**Fig. 1:**
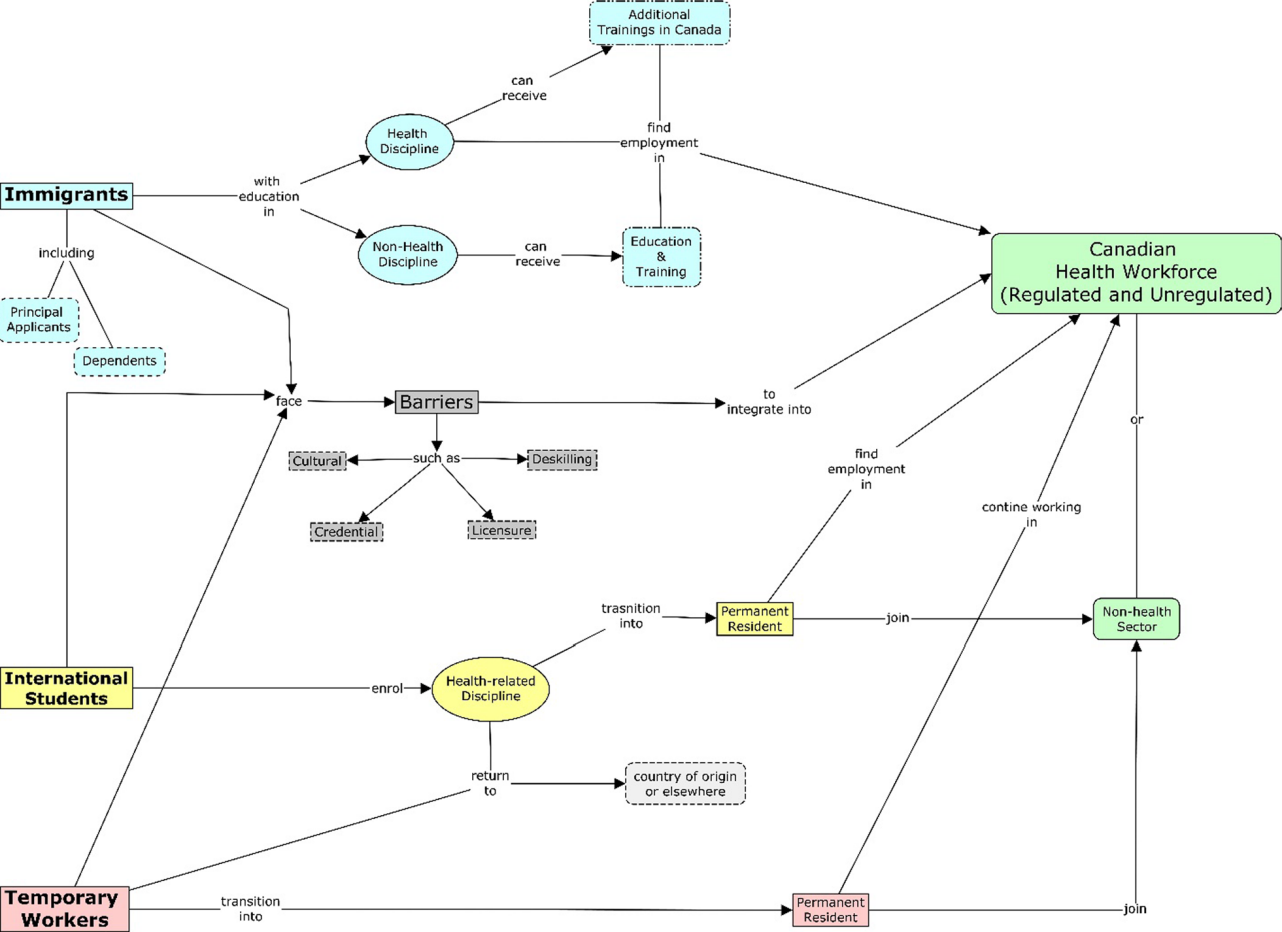
Pathways of immigrant integration into the Canadian health workforce

Evaluating immigrant participation in nursing and health care occupations, or the health workforce, in general, is a complex and data-intensive process. In part, this complexity reflects the multiple pathways immigrants can use to enter Canada and the health and care workforce, which is schematically presented in Fig. 1.

The figure shows three pathways into the Canadian health workforce for immigrants. First, immigrants can find employment in the Canadian health workforce depending upon their qualifications and the credential recognition process [14], they may also seek alternative careers in health or non-health-related occupations [2]. The second pathway is via the study–work visa route [24, 32]. International student enrolment in health-related disciplines in Canada has grown substantially, and about 30 percent of international students to Canada transition into permanent residence within 10 years of their first arrival [11]. The third pathway is temporary workers, of which there were about 470,000 in Canada in 2019 [19]. During the global COVID-19 pandemic the Canadian government allowed temporary workers (including asylum seekers) who had been working in the health sector to apply for permanent residence [30].

Immigrant integration and success in the workforce is constrained by several factors, including language and communication issues, workplace integration issues, inequality in opportunities, credentialing difficulties, and differences in occupational practices between the origin and destination countries [3, 20]. These factors can contribute to immigrant health worker deskilling and stratification into roles below their training level [13, 25, 33]. Previous studies also reveal the underutilization of immigrant workers in Canada’s health care sector, with 47 and 33 percent underutilization reported for foreign-educated immigrants and Canadian educated immigrants, respectively [17].

**Fig. 2:**
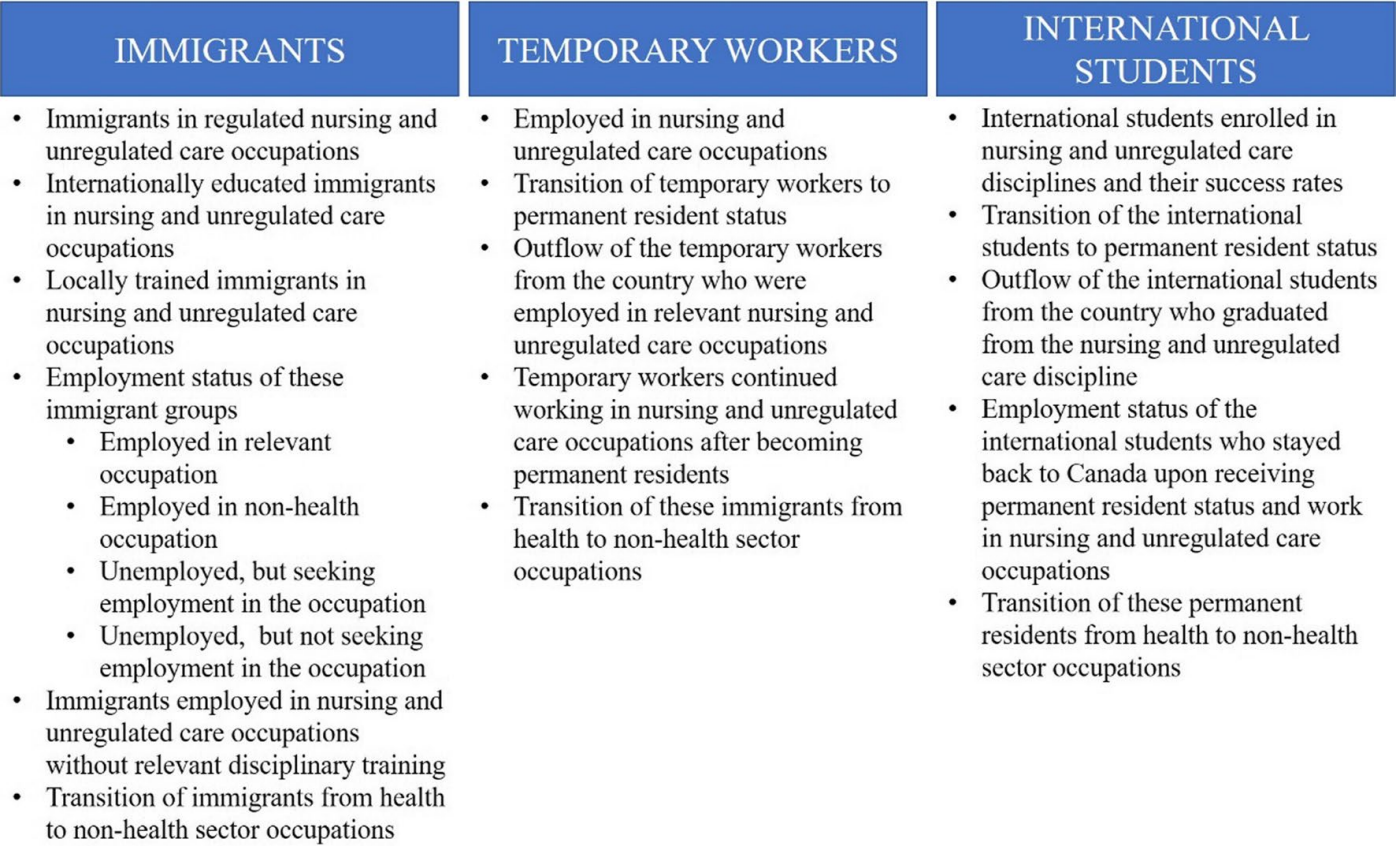
Information required for enumerating immigrants in nursing and unregulated care occupations

Figure 2 provides a brief idea of the data needed to comprehensively evaluate the contribution of immigrants to the health and care workforce in Canada. The educational qualifications and backgrounds, employment conditions, out-migration rates, and demographic characteristics of these groups are critical, and analysis at different spatial scales must be possible for improved health workforce planning.

At present, there is no readily available dataset that can enumerate immigrants in nursing and health care support occupations. While the Canadian population census, the Longitudinal Immigration Database (IMDB), and Labor Force Survey (LFS) available from Statistics Canada offer potential resources, these datasets come with availability, comprehensiveness, and administrative challenges. For example, census data are only available every 5 years, and the IMDB and LFS do not contain variables that can help identify immigrants who are employed in nursing and health care related occupations. Among other data sources is the CIHI, which is of limited use since it does not record citizenship status or ethnicity of health workers, and only contains information on certain regulated health occupations [9]. All the data mentioned above are also limited by the scale they report on, involve data privacy issues, and require fees for access.

## Methodology

We use descriptive analysis of CIHI health workforce data, including publicly available aggregate-level data (from 2010 to 2019), and a special customized dataset on IENs in Ontario (2011 and 2020) [10]. Additional customized population census data on immigrants in nursing and care-related occupations were acquired for the year 2016 [28]. Visa issuance and intended occupations of landed permanent residents for the years 2015 to 2020 was accessed through Immigration, Refugees, and Citizenship Canada open government portal [16]. Using this combination of data sources, we provide a multi-scalar geographic focus, including Canada, Ontario and select LHINs.

## Canada’s nursing workforce and immigrants

Regulated nurses in Canada include three categories, Nurse Practitioners (NPs), Registered Nurses (RNs) and Registered Practical Nurses (RPNs)/Licensed Practical Nurses (LPNs), each of which differs in their scope of practice. NPs are advanced practice registered nurses who provide direct care, including diagnosis and management of diseases, in some provinces they can prescribe medication, order lab tests, and provide specialist referrals. RNs provide care services and support clients in managing health issues ranging from illness, injury, to disability. The RPNs/LPNs assess clients and work in health promotion and illness prevention. Although the responsibilities of RPNs/LPNs and RNs may overlap, the scope of practice of RNs exceeds that of RPNs/LPNs [18].

Regulated nurses are the largest single occupational group in the Canadian health care workforce. In 2019, they represented 48.53 percent of total health workers in the country (438,222 of 902,900). In 2019 in the three major Canadian provinces—Ontario, British Columbia, and Quebec—regulated nurses represented 48.13, 48.28, and 50.44 percent of their respective health workforces. The highest representation was observed in Prince Edward Island and Newfoundland and Labrador, respectively, representing 60 and 55 percent of their total health workforce. Alberta registered the lowest share (43.76 percent).

The top and bottom nursing cadres, i.e. NPs and RPNs/LPNs, are growing at a much higher rate compared to RNs. As summarized in Table 1, NPs registered a 41.83 percent (4303 vs 6103) growth between the two years, and the equivalent number for LPNs/RPNs and RNs were, respectively, 11.90 (113,157 vs 126,620) and 2.93 percent. This pattern is evident at the provincial scale in most cases, but not all. For example, BC, QC, and SK, manifested smaller growth in LPNs compared to RNs between the two years. Regardless, on an average, the growth of RNs was much lower across provinces compared to RPNs/LPNs and that of NPs was consistently higher.

**Table 1:** Number of nurses in Canada's provinces by cadre in 2015 and 2019

	ON		QC		BC		SK		AB		NB		PEI		NL		NS		MB	
	2015	2019	2015	2019	2015	2019	2015	2019	2015	2019	2015	2019	2015	2019	2015	2019	2015	2019	2015	2019
All regulated nurses	148,666	160,137	99,059	101,233	49,915	53,733	14,793	15,678	48,816	53,340	11,628	11,444	2,247	2,489	8,448	8,344	13,581	14,030	16,984	17,794
Nurse practitioners	2520	3451	305	545	315	514	186	236	414	571	109	139	17	43	136	183	149	204	152	217
Registered nurses	102,490	103,877	70,042	72,695	35,397	38,041	10,226	10,940	34,764	35,907	8240	8019	1586	1704	6008	5771	9524	9508	12,634	12,995
Licensed practical nurses	43,656	52,809	28,712	27,993	11,619	12,351	3503	3700	12,330	15,513	3279	3286	644	742	2304	2390	3908	4318	3202	3518

**Fig. 3:**
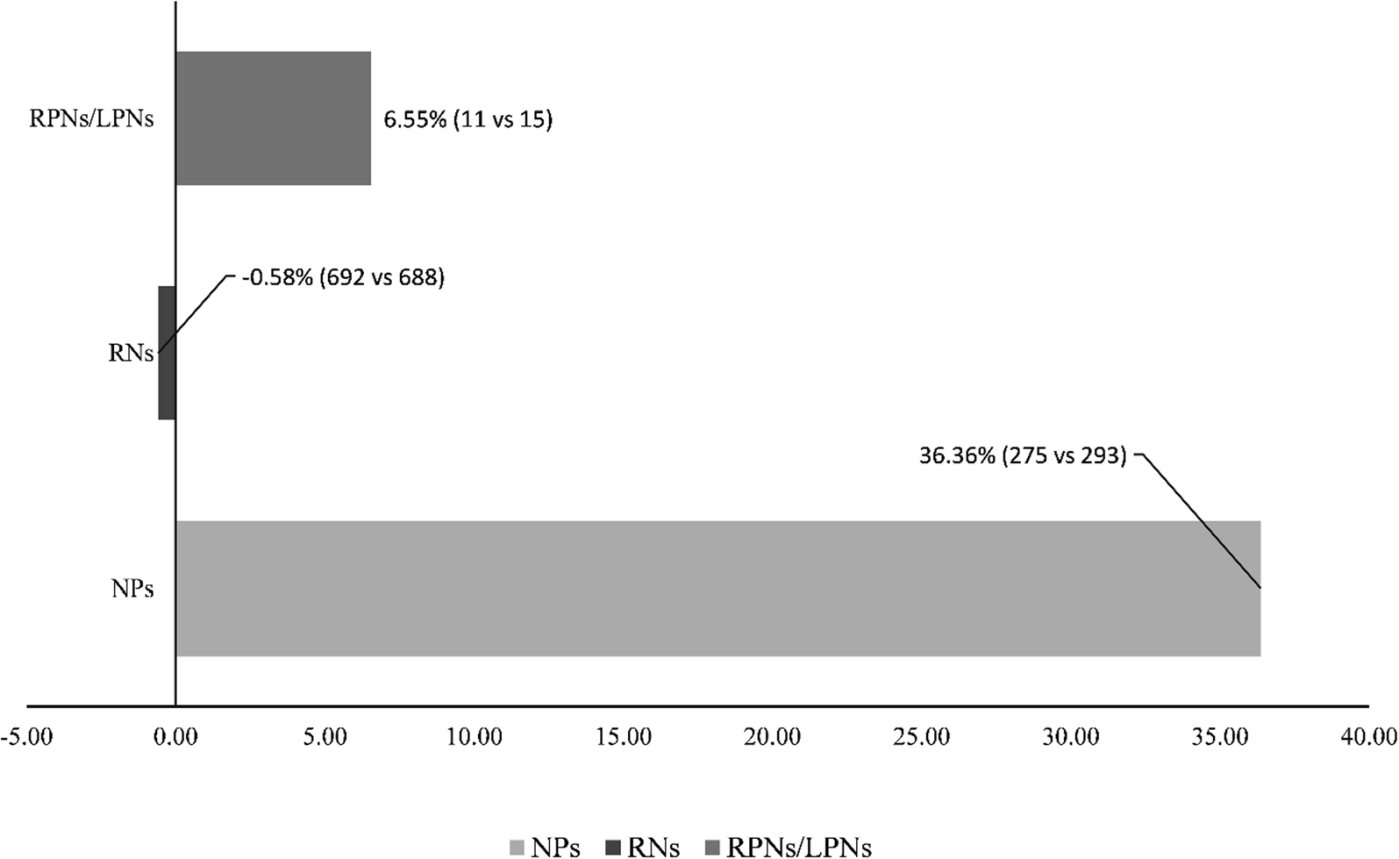
Direct Care Nurses rate of growth per 100,000 population in Canada in 2019 (CIHI Health Workforce Datasets)

RNs also registered a negative growth when evaluated relative to population size. As shown in Fig. 3, RNs declined per 100,000 population between 2015 and 2019, whereas the growth in NPs and RPNs/LPNs grew 36.36 and 6.55 percent, respectively. This pattern is consistent with an hourglass shape of nursing cadre growth in Canada where the top and bottom cadres experience growth, while the middle RN cadre experiences relative decline in relation to population growth.

Immigrants are entering this changing nursing workforce and contribute to these distributional shifts across the cadres. In 2016, immigrants represented 28 percent of the total population employed in nursing and health care occupations [12].^2^ The sharpest growth in the proportion was among the RPNs/LPNs (9 percent in 1996 vs 21.2 percent in 2016). Immigrants working in nursing and health care support occupations are mostly female. In 2016, Ontario had 60,135 adult immigrants employed in nursing and health care support occupations, and about 53,860 of them were females, representing 91 percent of the total employed immigrants in the occupation. Unfortunately, the gender distribution of immigrants by nursing cadre could not be determined in our data due to data privacy issues.

## Immigrants in Ontario’s nursing workforce

CIHI records information on the three nursing cadres by graduation location and includes IENs. Table 2 summarizes the number of IENs in Ontario by cadre in 2011 and 2020. Data show that the number of IENs working as RNs and NPs increased from 11,230 to 11,550 between 2011 and 2020, a 2.65 percent growth.^3^ Internationally educated RPNs manifested a substantially higher growth of 255.2 percent (from 1587 to 5637).

**Table 2:** Immigrant nurses in Ontario by cadre in 2011 and 2020

Place of work	RNs and NPs			RPNs		
	2011	2020	Change (%)	2011	2020	Change (%)
Hospital	6881	7318	6.35	379	1533	304.49
Community health	1285	1351	5.14	178	1177	561.24
Nursing home/Long-term care	1677	2038	21.53	880	2561	191.02
Other	760	841		53	363	
Not stated	627	2		97	3	
Total	11,230	11,550	2.85	1587	5637	255.20

In terms of place of work, long-term care (LTC) facilities registered the highest growth of NP/RN IENs, increasing by 21.53 percent between 2011 and 2020. RPN IENs increased by 255.20 percent over the same period, with the majority working in LTC. IEN sources of origin in Ontario was led by the Philippines with 3097 RNs/NPs and 2575 RPNs, followed by India, with 2275 RNs and NPs, and 1839 RPNs in 2020.

## IENs in Ontario’s LHINs

Table 3 summarizes the distribution of IENs by cadre across Ontario’s LHINs. The largest number of IENs was employed in the Toronto Central LHIN in 2020 as RNs and NPs, representing 27.22 percent of the total for the group in Ontario. Some locations with high populations, such as the Toronto Central LHIN, experienced a decline in the number of IENs, whereas some mid-sized cities saw an increase. For example, there were 3869 RN/NP IENs in 2011 in Toronto Central LHIN, which declined by 18.74 percent to 3144 in 2020. In contrast, the group experienced a substantial growth in mid-size LHINs like Waterloo Wellington (59.74 percent) and Southwest (63.94 percent) (Table 4). This trend may reflect the increasing decentralization of migration governance that has been occurring in Canada to encourage redistribution of immigrants away from the largest metropolitan centres [27].

**Table 3:** Spatial distribution of IENs in Ontario's LHINs

	RNs including NPs					RPNs				
	2011		2020		Change between 2011 and 2020	2011		2020		Changes between 2011 and 2020
	Count	%	Count	%	%	Count	%	Count	%	%
Erie St. Clair LHIN	184	1.64	161	1.39	− 12.50	34	2.14	61	1.08	79.41
South West LHIN	330	2.94	541	4.68	63.94	39	2.46	359	6.37	820.51
Waterloo Wellington LHIN	231	2.06	369	3.19	59.74	54	3.40	240	4.26	344.44
Hamilton Niagara Haldimand Brant LHIN	780	6.95	1007	8.72	29.10	146	9.20	591	10.48	304.79
Central West LHIN	691	6.15	852	7.38	23.30	96	6.05	389	6.90	305.21
Mississauga Halton LHIN	1203	10.71	1379	11.94	14.63	227	14.30	775	13.75	241.41
Toronto Central LHIN	3869	34.46	3144	27.22	− 18.74	353	22.24	1086	19.27	207.65
Central LHIN	1736	15.46	1805	15.63	3.97	329	20.73	1079	19.14	227.96
Central East LHIN	1219	10.86	1133	9.81	− 7.05	177	11.15	494	8.76	179.10
South East LHIN	98	0.87	119	1.03	21.43	10	0.63	47	0.83	370.00
Champlain LHIN	571	5.09	644	5.58	12.78	78	4.91	327	5.80	319.23
North Simcoe Muskoka LHIN	95	0.85	126	1.09	32.63	21	1.32	94	1.67	347.62
North East LHIN	147	1.31	158	1.37	7.48	11	0.69	49	0.87	345.45
North West LHIN	74	0.66	112	0.97	51.35	12	0.76	46	0.82	283.33
Total	11,228	100.00	11,550	100.00	2.87	1587	100.00	5637	100.00	255.20

**Table 4:** IENs from the Philippines and India in Ontario's LHINs in 2011 and 2020

	Philippine graduate RNs and NPs			India graduate RNs and NPs			Philippine graduate RPNs			India graduate RPNs		
	2011	2020	Change (%)	2011	2021	Change (%)	2011	2020	Change (%)	2011	2020	Change (%)
Erie St. Clair LHIN	41	32	− 21.95	13	23	76.92	8	18	125.00	†	21	
South West LHIN	31	40	29.03	16	255	1,493.75	9	78	766.67	†	228	
Waterloo Wellington LHIN	20	30	50.00	19	139	631.58	5	74	1,380.00	6	109	1,716.67
Hamilton Niagara Haldimand Brant LHIN	164	215	31.10	42	238	466.67	32	243	659.38	12	229	1,808.33
Central West LHIN	168	152	− 9.52	239	425	77.82	15	58	286.67	31	276	790.32
Mississauga Halton LHIN	355	377	6.20	115	298	159.13	64	302	371.88	45	310	588.89
Toronto Central LHIN	1403	1185	− 15.54	186	243	30.65	126	696	452.38	17	149	776.47
Central LHIN	514	522	1.56	110	220	100.00	85	585	588.24	25	229	816.00
Central East LHIN	402	363	− 9.70	110	220	100.00	62	233	275.81	12	145	1108.33
South East LHIN	13	18	38.46	10	37	270.00	†	24		0	12	
Champlain LHIN	78	114	46.15	26	73	180.77	20	191	855.00	7	59	742.86
North Simcoe Muskoka LHIN	11	14	27.27	†	44		6	39	550.00	†	31	
North East LHIN	17	19	11.76	†	20		†	20		†	19	
North West LHIN	9	16	77.78	†	40		†	14		0	22	
Total	3226	3097	− 4.00	890	2275	155.62	441	2575	483.90	161	1839	1042.24

Although the highest representation of RPN IENs was in Toronto Central LHIN, followed by the Central and Mississauga Halton, it was the Southwest LHIN that experienced the highest growth between 2011 and 2020, with an 820 percent increase in the number of RPN IENs. The Waterloo Wellington LHIN also experienced significant growth (344 percent) of RPN IENs. In the case of RPN IENs from the Philippines, all the LHINs have witnessed considerable growth between 2011 and 2020, especially the Waterloo Wellington, Southwest, Toronto Central, and Hamilton Niagara LHINs. Moreover, the growth of IENs from India was much higher than that of Philippines and is rapidly growing in all three nursing cadres. The larger increase in IENs as RPNs compared to RNs requires further examination, but likely reflects how previous education is assessed by the College of Nurses of Ontario (the professional regulator). Diploma program graduates are eligible to apply as RPNs and baccalaureate degree graduates as RNs, and each of these cadres completes a different qualifying exam.^4^

## Immigrants in health care support occupations other than nursing

Immigrant participation in the health and care sector also includes unregulated care occupations, nursing aides, orderlies, and patient associates. In 2016, about 245,500 people were employed as nurse aides, orderlies and patient service associates in Canada, of which one third were immigrants, many of whom had some prior health education [31].^5^

Personal Support Workers (PSW) are an example of such occupational groups. PSWs work alongside and under the supervision of regulated nurses in institutional settings and aid patients with various daily living activities, and perform nursing services delegated by regulated nurses in Ontario [1]. During the COVID-19 pandemic, the role of PSWs in Ontario’s LTC became a significant policy focus in Ontario [15, 21].

**Fig. 4:**
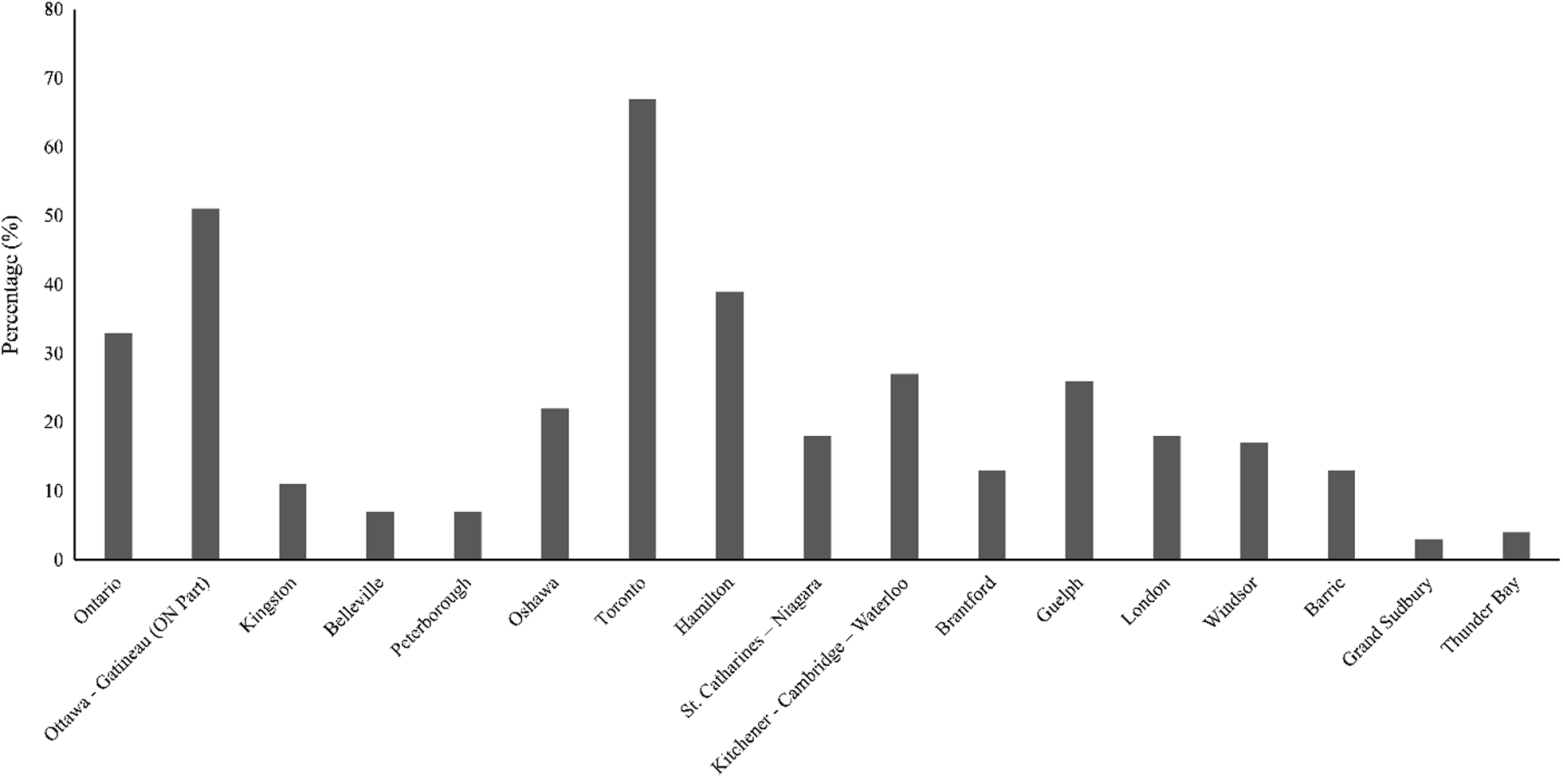
Immigrants as nursing aides, orderlies, and patient service associates by CMA in 2016 (Statistics Canada [28])

**Fig. 5:**
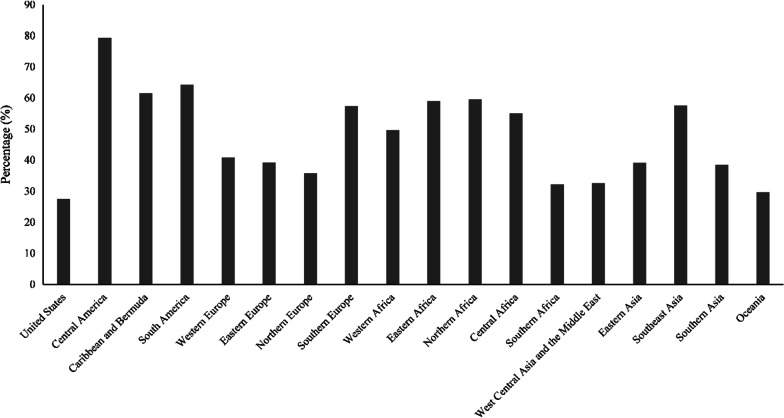
Origins of nursing aides, orderlies, and patient service associates in Ontario in 2016 (Statistics Canada [28])

Mattison and Lavis [22] report that the Ministry of Health and Long-term Care estimated nearly 100,000 PSWs in Ontario. Approximately 57,000 were employed in LTC homes, over 34,000 in home and community care, and about 7,000 in hospitals [23]. Figure 4 presents the spatial distribution of immigrants in nursing aides, orderlies, and patient service associate occupations in Ontario by metropolitan region. Figure 5 shows the total number of immigrants relative to their totals in professional nursing occupations. The data emphatically point towards a significantly higher representation of immigrants from Western Africa, Southern Europe, Northern Africa, Central Africa, Eastern Africa, Southeast Asia, Caribbean and Bermuda, South America, and Central America.

## Prospects of immigrants in nursing and health care support occupations

Table 5 summarizes the intended occupations of permanent residents in selected nursing and health care support services in Ontario. The data point toward a high intention of immigrants to work in nursing and health care-related services upon arrival. About 1,025 permanent residents expressed interest in working in any of the three nursing and care-related occupations from 2015 to 2020.

**Table 5:** Intended occupation of landed immigrants in Ontario from 2015 to 2020 ( source: Statistics Canada (2021b) [29].)

Occupations	2015	2016	2017	2018	2019	2020	Total
Registered nurses and registered psychiatric nurses	270	170	130	135	50	20	775
Registered practical nurses	30	5	15	15	10	–	75
Nurse aides, orderlies, and patient service associates	60	35	25	20	20	15	175
Total	360	210	170	170	80	35	1025

Meanwhile, data from Table 6 summarize the number of study permits and renewals in Canada by academic discipline, revealing an increase in the number of international students in health-related disciplines between 2015 and 2019. Data indicate a higher growth of international students in health sciences compared to other disciplines, with a 129 percent increase (from 7554 to 17,338).

**Table 6:** Study permit issuance and renewal of international students in Canada ( source: IRCC 2021)

Area of study	2015	2019	Growth (%)
Medicine	812	1027	26
Applied sciences	21,730	31,692	46
General sciences	10,808	17,189	59
Health sciences	7554	19,338	129

The data highlight the significant potential of immigrants to participate in Canada’s nursing and health care support occupations, but understanding the full extent of this is limited by available data.

## Conclusion

This article has provided a multi-scalar review of immigrants’ employment in nursing and health care support occupations in Canada, Ontario and select LHINs therein with reference to the milestones the WHO’s Global Strategy on HRH recommend. To assess the contribution of immigrants to Canada’s health workforce, we outline the diverse pathways used, and barriers immigrants face in entering the health workforce, particularly regulated health professions such as nursing.

Having indicated some of the data challenges that accompany the complexity of pathways, we then outlined the methodology of this paper in terms of which data, we were able to access to determine the potential and actual contribution immigrants make  to Canada’s health care sector. We reviewed numbers related to Canada’s nursing workforce, revealing the changing relative growth of NPs, RNs and RPNs/LPNs. Nationally data indicate a growth in the upper and lower nursing cadres and a squeeze on the number of RNs in the middle. Across Canada provincial numbers indicate a reduction in the ratio of RNs to population numbers. Immigrants have been entering this nursing labour force as IENs primarily through the RPN/LPN cadre, and assessing the contribution of IENs to these shifts in nursing cadres poses an important ongoing research agenda. The source of immigrants has also shifted away from some traditional locations (such as the UK), to India, and the Philippines.

Looking at Ontario data, we see disproportionate numbers of IENs working in the largest urban LHINs, but larger increases are registered across mid-sized communities. The increased presence of IEN RPNs is evident across the majority of LHINs, as is the increase in IENs coming from India and the Philippines. The number of RN and RPN IENs have increased most notably in LTC and community-based health. Understanding the degree of ethnic diversity evident in workplaces and the interactions and experiences of IENs must be captured in appropriate data bases.

Immigrant workers also make an important contribution to unregulated PSW and care aide positions that increasingly support care delivery in LTC and home and hospital-based care. These workers increasingly collaborate with regulated health professions, but there is evidence that immigrants with health training and credentials (including nursing) are working in these unregulated occupations [31]. This echoes the excess underutilization seen in immigrants with health care backgrounds working in Canada, especially compared to Canadian born workers [17]. Effective assessment of this process will permit policy responses to be developed, as well as register Canada’s responsibility to prevent the underutilization of skills that internationally trained health care workers bring to the country. Canadian policy makers must also consider the consequences of these processes for health systems in sending nations, in line with the spirit of the WHO Code.

This paper indicates the importance of including immigrants in any accounting of Canada’s HRH strategy. Immigrant workers make a significant contribution to health occupations across the country and across cadres. Although the immigrants working in nursing and health care support occupations are mostly female, the information we used cannot be gender disaggregated by specific nursing cadres due to data privacy issues. We focused specifically on nursing and care aide workers, revealing the multiple pathways now used to enter Canada and the health and care workforce. This complexity contributes to the difficulty of accessing timely, relevant, and integrated data on the health workforce in Canada. The need for better HRH data has become more evident considering the consequences of the global pandemic and as calls for a national workforce strategy have become louder.^6^ Integrated data bases on the health workforce must include immigrant workers, including those in regulated and unregulated allied occupations. Understanding how Canada uses and incorporates immigrant HRH into the health sector is necessary for effective workforce planning, but also for assessing Canada’s impact on global health workforce distribution and utilization.

## Data Availability

The data used in this research can be readily accessed through “Canadian Institute for Health Information (CIHI)” website for free. Part of the research uses customized individual-level data procured from CIHI. For more information on the data, please contact the authors.

## Abbreviations

CIHI: Canadian Institute for Health Information
HRH: Health human resources
IENs: Internationally Educated Nurses
LHINs: Local Health Integration Networks
LPNs: Licensed Practical Nurses
LTC: Long-term care
NPs: Nurse Practitioners
PSWs: Personal Support Workers
RNs: Registered Nurses
RPNs: Registered Practical Nurses
WHO: World Health Organization

## Footnotes

1 LHINs are crown agencies established by the Ontario government for local-level planning, coordination, integration, and funding of health care service providers, including hospitals, community health centres, long-term care services, mental health and addiction agencies, and community support service agencies.

2 Nursing and health care support occupations, as defined by Cornelissen [12] included registered nurses and registered psychiatric nurses, licensed practical nurses, and nursing aides, orderlies, and patient service associates.

3 Because of data privacy issues, RNs and NPs were grouped together by CIHI when delivering the customized data.

4 https://www.cno.org/en/become-a-nurse/new-applicants1/outside-canada/examinations/.

5 Nurse aides, orderlies, and patient service associates include occupations, such as health care aid, long-term care aide, nurse aide, nursing attendant, patient care aide, personal care aide, resident care aide, and excludes LPNs and professional occupations in nursing.

6 See https://www.hhr-rhs.ca/en/petition.html for a national call to action to significantly enhance existing health workforce data infrastructure in Canada.
